# Responses to Real-World and Hypothetical E-Cigarette Flavor Bans Among US Young Adults Who Use Flavored E-Cigarettes

**DOI:** 10.1093/ntr/ntad258

**Published:** 2023-12-23

**Authors:** Jamie Tam, Evelyn Jimenez-Mendoza, John Buckell, Jody Sindelar, Rafael Meza

**Affiliations:** Department of Health Policy and Management, Yale School of Public Health, New Haven, CT, USA; Department of Epidemiology, University of Michigan School of Public health, Ann Arbor, MI, USA; Health Economics Research Centre, Nuffield Department of Population Health, University of Oxford, Oxford, UK; Department of Health Policy and Management, Yale School of Public Health, New Haven, CT, USA; Department of Integrative Oncology, BC Cancer Research Institute, BC Cancer Research Institute, Vancouver, British Columbia, Canada; School of Population and Public Health, University of British Columbia, Vancouver, British Columbia, Canada

## Abstract

**Introduction:**

E-cigarette flavor bans could reduce or exacerbate population health harms. To determine how US e-cigarette flavor restrictions might influence tobacco use behavior, this study assesses responses to real-world and hypothetical flavor bans among young adults who use flavored e-cigarettes.

**Aims and Methods:**

An online, national survey of young adults ages 18–34 who use flavored e-cigarettes was conducted in 2021 (*n* = 1253), oversampling states affected by e-cigarette flavor restrictions. Participants were asked about their responses to real-world changes in the availability of flavored e-cigarettes. Unaffected participants were asked to predict their responses under a hypothetical federal e-cigarette flavor ban.

**Results:**

The most common response to real-world changes in flavored e-cigarettes availability was to continue vaping (~80%). Among those who exclusively vaped, 12.5% switched to combustible tobacco. Quitting all forms of tobacco was selected by 5.3% of those exclusively vape versus 4.2% who dual use. Under a hypothetical federal ban, more than half of respondents stated they would continue vaping; 20.9% and 42.5% of those who exclusively vape versus dual use would use combustible tobacco. Quitting all tobacco products was endorsed by 34.5% and 17.2% of those who exclusively vape versus dual use.

**Conclusions:**

Young adults who vape flavored e-cigarettes have mixed responses to e-cigarette flavor bans. Under both real-world and hypothetical e-cigarette flavor bans, most who use flavored e-cigarettes continue vaping. Under a real-world ban, the second most common response among those who exclusively vape is to switch to smoking; under a hypothetical federal ban, it is to quit all tobacco.

**Implications:**

This is the first national survey to directly ask young adults who use flavored e-cigarettes about their responses to real-world changes in flavored e-cigarette availability due to state and local flavor restrictions. The survey also asked individuals to predict their responses under a hypothetical federal e-cigarette flavor ban. Most who use flavored e-cigarettes would continue vaping following e-cigarette flavor restrictions, but many would switch to or continue using combustible tobacco, highlighting potential negative public health consequences of these policies. Policymakers must consider the impact of e-cigarette flavor bans on both e-cigarette and cigarette use.

## Introduction

Major federal actions on flavored e-cigarettes are occurring: the US FDA is reviewing applications from e-cigarette manufacturers and determining which products—and which flavors—will be allowed to stay on the market.^1^ To date, no flavored e-cigarettes, other than tobacco-flavored, have received marketing authorizations from the FDA. If none receive authorization, this becomes a de facto federal ban on all non-tobacco flavored e-cigarette products.

Local and state restrictions on flavored e-cigarettes could influence switching between e-cigarettes and cigarettes because the two products may be substitutes.^2–5^ Flavor restrictions can reduce health risks if those who use e-cigarettes respond by quitting all tobacco. Conversely, if people who vape exclusively respond by switching to cigarettes, this would exacerbate health risks.^6^ Quasi-experimental analyses show that e-cigarette flavor restrictions increase cigarette sales.^5^ A recent review identified studies evaluating behavioral responses to both real-world and hypothetical e-cigarette flavor bans.^7^ Most studies of real-world e-cigarette flavor restrictions use youth data,^8–13^ but some use adult data.^14,15^ The real-world effects of bans on young adults require further examination. Online experiments indicate that e-cigarette flavor restrictions may result in increased cigarette smoking.^3,4^ Other studies surveyed US adults who use tobacco about their potential reactions if a flavor restriction was implemented.^16,17^

Among surveys asking about hypothetical bans, respondents are typically allowed to select only one behavioral response. However, under a federal flavor ban, people who use e-cigarettes may engage in multiple behaviors depending on the ease with which they could continue acquiring flavored products, that is, through an illicit market. To assess both potential public health benefits and harms associated with such a ban, this study uses a national online survey of young adults who use flavored e-cigarettes to assess the full range and distribution of responses to real-world flavor restrictions or hypothetical flavor ban scenarios.

## Methods

An online national survey of young adults who use flavored e-cigarettes ages 18–34 was conducted in 2021 (*n* = 1253). Respondents in states with flavor restrictions were oversampled to obtain a sufficiently large sample who could report real-world responses. See Table S1–S3 for state selection and sample characteristics.

Participants were queried about their tobacco use prior to Thanksgiving 2019, before most state flavor restrictions were implemented. Thanksgiving 2019 was used as an anchor to reduce risk of recall bias. Those who exclusively used e-cigarettes reported vaping on ≥7 of the 30 days before Thanksgiving 2019 but did not smoke. Those who dual used e-cigarettes with cigarettes reported smoking on ≥1 of 30 days before Thanksgiving 2019 and had smoked at least 100 cigarettes in their lifetime. Participants were then asked if at some point between Thanksgiving 2019 and the time of the survey, they were unable to buy flavored e-cigarettes as usual. Respondents who were affected were assigned to the real-world flavor restriction survey questions (*n* = 750); those not affected must have vaped on ≥7 of the past 30 days at time of survey and were asked how they would respond to a hypothetical federal policy (*n* = 503; Figure S1).

### Real-World Flavored E-Cigarette Restrictions

We operationalized the real-world effect of a “ban” as the inability to purchase flavored e-cigarettes from a retail store. Only participants who stated that their inability to purchase was due to flavored e-cigarettes being unavailable were asked the follow-up question: “After this change when you could not purchase flavored e-cigarettes, what did you do? Select all that apply.” Response options included quitting all tobacco use, continuing to vape flavored e-cigarettes, and switching to tobacco flavored e-cigarettes, cigarettes or other combustible tobacco, smokeless tobacco, or heated tobacco.

Those who continued vaping flavored e-cigarettes were asked: “How were you able to continue obtaining flavored e-cigarettes? Select all that apply.” with options for purchasing from the black market, from another jurisdiction, online, or obtaining through family and friends, modifying their e-cigarettes, or other.

### Hypothetical Flavored E-Cigarette Ban

Individuals who use e-cigarettes and were unaffected by real-world flavor restrictions were asked, “Suppose the federal government bans sales of all flavored e-cigarettes in the United States and you are no longer able to purchase them through your usual source. What do you think you would do? Select all that apply.”

Those who stated that they would continue using flavored e-cigarettes were similarly asked: “How would you try to continue obtaining flavored e-cigarettes?” with the same response options as above.

We calculated the weighted proportion of individuals reporting each of the real-world or hypothetical ban outcomes. Individual responses were weighted by the inverse of the population prevalence of each survey quota group. We separately examine responses from people who exclusively use e-cigarettes versus dual use.

## Results

Tobacco use responses to bans were categorized into four types: combustible tobacco use (C), heated or smokeless tobacco use (S), e-cigarette use (E), and quitting all forms of tobacco (Q). For both real-world and hypothetical bans, most individuals selected only one response option.

Table 1 presents responses to real-world e-cigarette flavor restrictions among people who use e-cigarettes. In total 80.9% of all respondents continued to use e-cigarettes (see Supplement for combined data). Among these, 60.2% continued vaping flavored e-cigarettes while 25.9% switched to non-flavored e-cigarettes; 13.9% used both (not shown). Some who vape exclusively (12.5%) and dual use (38.6%) responded by using combustible tobacco. The third most common response among exclusive users was to quit all forms of tobacco (5.3%) and the least common response was to use heated or smokeless tobacco (3.1%). Among dual users, this was inverted, 14.6% used heated or smokeless tobacco, and 4.2% quit all forms of tobacco. In comparison to 50.0% of people who dual use, 79.7% of people who vape exclusively chose to continue vaping as their only response to flavor restrictions. Less than 2.6% of respondents reported quitting all forms of tobacco as their only response.

**Table 1.** Responses to Real-World E-Cigarette Flavor Restrictions Among US Young Adults Who Use Flavored E-Cigarettes

Young adults who use flavored e-cigarettes, *n* = 750		Responses to ban				Young adults who use e-cigarettes exclusively, *n* = 281			Young adults who dual use, *n* = 469		
		C	S	E	Q	n	%		n	%	
Any combustible tobacco use, *n* = 204 (27.3%)		✓				17	7.8	*N* = 31 (12.5%)	63	17.5	*N* = 173 (38.6%)
		✓	✓			1	0.3		11	2	
		✓		✓		12	4.3		67	14.1	
		✓	✓	✓		1	0.2		23	3.6	
		✓		✓	✓	0	0		3	0.6	
		✓	✓		✓	0	0		1	0.1	
		✓	✓	✓	✓	0	0		3	0.3	
		✓			✓	0	0		2	0.4	
Any heated or smokeless tobacco use, but no combustible tobacco use, *n* = 49 (6.0%)			✓			4	1.5	*N* = 9 (2.6%)	10	1.5	*N* = 40 (8.5%)
			✓	✓		4	0.9		30	7	
			✓		✓	1	0.2		0	0	
			✓	✓	✓	0	0		0	0	
Continued vaping only, or quit all tobacco, *n* = 473 (64%)				✓		223	79.7	*N* = 226 (80.5%)	242	50	*N* = 247 (51.4%)
				✓	✓	3	0.9		5	1.4	
Quit all tobacco, *n* = 24 (2.6%)					✓	15	4.3	*N* = 15 (4.3%)	9	1.4	*N* = 9 (1.4%)
Young adults who use e-cigarettes exclusively	n*	31	11	243	19						
	%*	12.5	3.1	85.9	5.3						
Young adults who dual use	n*	173	78	373	23						
	%*	38.6	14.6	77.1	4.2						

Survey respondents who reported being affected by state or local flavor restrictions were asked, “After this change when you could not purchase flavored e-cigarettes (eg, menthol/mint, fruit, candy, others), what did you do? Select all that apply.” C = switch to smoking cigarettes or other combustible tobacco (eg, cigars, hookah, pipe tobacco, bidis); S = switch to using smokeless tobacco (e.g., chewing tobacco, snus, snuff, dip, dissolvables) or heated tobacco (e.g., IQOS, eclipse); E = switch from JUUL to other flavored e-cigarettes or switch to using tobacco-flavored e-cigarettes or continued vaping flavored e-cigarettes by getting them from a different source; Q = quit all vaping and tobacco use. Each row represents one of 15 possible response combinations: gray boxes with check marks represent the selected response for each product category, while dark gray boxes highlight those who only reported a single response type. Each column represents any response combination that included the specific product (C, S, E, Q); *numbers do not sum to 100% of the sample because categories are not mutually exclusive.

Table 2 shows responses to a hypothetical federal e-cigarette flavor ban among people who use flavored e-cigarettes. “Continue e-cigarette use” was the most common response reported by most who vape exclusively (60.8%) and dual use (60.4%): among these, 42.7% would vape flavored e-cigarettes; 37.1% would use non-flavored e-cigarettes; 20.2% stated they would use both (not shown); 34.5% of those who exclusively vape would quit all forms of tobacco and 20.9% stated that they would use combustible tobacco in response to a hypothetical flavor ban. Among dual users, the second most common response to a hypothetical flavor ban was to use combustible tobacco (42.5%) followed by quitting all forms of tobacco (17.2%). The least common response among both groups was to use heated or smokeless tobacco (people who exclusively vape: 7.1% vs. dual use: 15.4%). Among those who only selected one response type, 7.9% of respondents who vape exclusively would switch to smoking, compared to 21.1% of those who dual use. Less than half of respondents (45.3%) who exclusively vape would only continue vaping, whereas 38.1% of those who dual use listed continued vaping as their sole response. Quitting was the sole response of 26.7% of individuals who vape exclusively and 11.6% of individuals who dual use. The use of heated or smokeless tobacco was the least endorsed response option.

**Table 2.** Responses to Hypothetical E-Cigarette Flavor Ban Among US Young Adults Who Use Flavored E-Cigarettes

Young adults who use flavored e-cigarettes, *n* = 503		Responses to ban				Young adults who use e-cigarettes exclusively, *n* = 158			Young adults who dual use, *n* = 345		
		C	S	E	Q	n	%		n	%	
Any combustible tobacco use, *n* = 171 (34%)		✓				11	7.9	*N* = 29 (20.9%)	71	21.1	*N* = 142 (42.5%)
		✓	✓			2	1.8		10	2.7	
		✓		✓		11	5.5		38	11	
		✓	✓	✓		1	2.6		14	5.5	
		✓		✓	✓	1	1.1		3	0.5	
		✓	✓		✓	0	0		1	0.1	
		✓	✓	✓	✓	1	0.4		4	0.8	
		✓			✓	2	1.5		1	0.7	
Any heated or smokeless tobacco use, but no combustible tobacco use, *n* = 30 (4.7%)			✓			3	1.3	*N* = 5 (2.2%)	10	1.9	*N* = 25 (6.3%)
			✓	✓		2	0.9		8	2.5	
			✓		✓	0	0		5	1.5	
			✓	✓	✓	0	0		2	0.4	
Continue vaping only, or quit all tobacco, *n* = 219 (43.8%)				✓		75	45.3	*N* = 81 (50.2%)	131	38.1	*N* = 138 (39.6%)
				✓	✓	6	4.9		7	1.6	
Quit all tobacco, *n* = 83 (17.5%)					✓	43	26.7	*N* = 43 (26.7%)	40	11.6	*N* = 40 (11.6%)
Young adults who use e-cigarettes exclusively	n*	29	9	97	53						
	%*	20.9	7.1	60.8%	34.5%						
Young adults who dual use	n*	142	54	207	63						
	%*	42.5	15.4	60.4	17.2						

Survey respondents were asked, “Suppose the federal government bans sales of all flavored e-cigarettes in the United States and you are no longer able to purchase them through your usual source. What do you think you would do? Select all that apply.” C = switch to smoking menthol cigarettes, non-menthol cigarettes, or other combustible tobacco (eg, cigars, hookah, pipe tobacco, bidis); S = switch to using smokeless tobacco (eg, chewing tobacco, snus, snuff, dip, dissolvables) or heated tobacco (eg, IQOS, eclipse); E = switch to vaping tobacco-flavored e-cigarettes or continue vaping flavored e-cigarettes by getting them from a different source; Q = quit all vaping and tobacco use. Each row represents one of 15 possible response combinations: gray boxes with check marks represent the selected response for each product category, while dark gray boxes highlight those who only reported a single response type. Each column represents any response combination that included the specific product (C, S, E, Q); *numbers do not sum to 100% of the sample because categories are not mutually exclusive.

Many who experienced real-world e-cigarette flavor restrictions stated that they continued vaping flavored e-cigarettes by purchasing them outside their jurisdiction (44.6%) or online (50.3%), obtaining them through family and friends (30.1%) or illicit sources (8.2%), or modifying their e-cigarettes by adding their own flavors (11.7%) (Table S4).

Under a hypothetical ban, most would purchase flavored e-cigarettes online (77.1%), followed by accessing through family and friends (45.5%). Others would modify their e-cigarettes (22.3%) or purchase from illicit sources (16.6%) or another country (32.1%).

## Discussion

This study assesses young adult responses to e-cigarette flavor restrictions by examining both (1) real-world responses for those who were likely affected by them, and (2) hypothetical responses for those who had not been affected.

This study differs from most prior surveys because participants could select multiple responses. We believe this more closely mimics real-world possibilities. Under real-world flavor restrictions, affected individuals who use tobacco have multiple options available—which are not mutually exclusive and may seemingly conflict with each other—including switching, quitting, and acquiring products through illicit sources. The flexible options reveal the degree of openness to different responses among young adults who vape flavored e-cigarettes, and provide a range for the distribution of plausible behavioral responses to flavor restrictions.

This is the first study to ask young adults directly about how they responded to real-world changes in the availability of flavored e-cigarettes. Most respondents (80.9%) continued vaping following flavor restrictions. Many were able to access flavored products in other jurisdictions or through personal connections, 12.5% of those who vape exclusively and 38.6% of those who dual use included combustible tobacco use as one of their responses, 7.8% of those who exclusively vaped switched completely to combustible tobacco.

As for a hypothetical federal ban, 60.4% of participants expect to continue vaping—far less than the 80% in the real-world scenario. Responses to local and state flavor restrictions may differ from behavior under federal policy due to the ease with which individuals are able to purchase their flavored products beyond state or local borders. Interestingly, our data suggest that people may overestimate the likelihood of purchasing from illicit sources under hypothetical (16.6%) vs real-world conditions (8.2%).

Gravely et al. estimated that under a hypothetical e-cigarette flavor ban, 54.0% of people who vape flavored e-cigarettes would continue vaping, 21.0% would switch to smoking, 9.2% would quit all tobacco,^17^ but 28.6% of people who exclusively use e-cigarettes and 50.9% of young adults who dual use would respond by using combustible tobacco, and 29.7% and 18.2% would consider quitting all tobacco. This suggests that larger shares of this population might consider quitting if flavored e-cigarettes were made inaccessible, but that larger shares might also consider switching to smoking.

### Strengths

Key advantages of the study are that we directly asked: (1) whether the person noticed the change in availability of their flavored products—a better indication that a flavor ban was implemented and enforced in their locality, (2) why they changed their behavior, and (3) whether that change can be attributed directly to the absence of flavored products at retail stores. Numerous write-in responses indicated that local and state flavor restrictions were the reason. Furthermore, while loss of retailer access to flavored e-cigarettes is not a perfect proxy for exposure to a flavor restriction, it achieves the same outcome as a flavor restriction itself.

### Limitations

This descriptive study did not use control conditions or experimental manipulations, which limits causal inference. This study is not nationally representative; states with flavor restrictions were oversampled. Information was assessed retrospectively, which may introduce recall bias. It is unclear how this would influence findings, as those who failed to recall a change in flavored e-cigarette access were directed to the hypothetical ban survey questions. We did not ask about former smoking status on Thanksgiving 2019; some respondents may have previously quit smoking, which could influence the likelihood of taking up smoking following flavor restrictions. Real-world flavor restrictions coincided with COVID-19 and highly publicized lung injuries connected to vaping THC^18^; many were under the mistaken belief that vaping nicotine would lead to such injury, potentially affecting responses to flavor restrictions. Our hypothetical data rely on the respondent’s ability to predict future behavior under scenarios that they have not yet experienced. Finally, specific results for people who dual use are puzzling at first glance: under real-world e-cigarette flavor restrictions, 38.6% would continue smoking; and 42.5% under a hypothetical ban. (Presumably 100% of people who dual use would keep smoking.) We attribute this to the question wording; people who dual use may have skipped the option to select “Switch to smoking cigarettes”—more appropriate for participants who exclusively vape.

E-cigarette flavor restrictions have mixed influences on young adults who use e-cigarettes. Some continue vaping; others were encouraged towards combustible tobacco. Future studies should evaluate e-cigarette flavor restrictions in the context of menthol bans on cigarettes and cigars.^19^ Although this study focuses on people who already vape, the public health impact of e-cigarette flavor restrictions depends on how they impact tobacco use initiation among those who do not use tobacco.

## Supplementary Material

Supplementary material is available at *Nicotine and Tobacco Research* online.

ntad258_suppl_Supplementary_Figures_S1_Tables_S1_S5

ntad258_suppl_Supplementary_Data

## Data Availability

The data underlying this article will be made available upon request to the authors.
