# Supplementary material for: Responses to Real-World and Hypothetical E-Cigarette Flavor Bans Among US Young Adults Who Use Flavored E-Cigarettes
Source: Nicotine Tob Res. 2023 Dec 23;26(8):1113–7. doi: 10.1093/ntr/ntad258 (PMC11260892; doi:10.1093/ntr/ntad258)
Supplement: ntad258_suppl_Supplementary_Figures_S1_Tables_S1_S5 [file ntad258_suppl_supplementary_figures_s1_tables_s1_s5.docx]

**SUPPLEMENT**

**Survey design and sample recruitment**

Survey data were collected using the Qualtrics survey and platform from March 4, 2021 through December 23, 2021. Qualtrics, a national online research survey firm, solicited individuals from their existing panel of participants. Upon completion of the survey, participants were directed to Qualtrics for payment according to the terms Qualtrics provided, which included options for a gift card, points programs, charitable contributions, and/or partner products or services. Participants had the option to take the survey by smartphone and/or computer, with most choosing to use a smartphone. The survey was initially piloted on ~50 respondents for testing purposes before finalized for wider release. To ensure activity during the survey, respondents were required to move a slider prior to proceeding to the next question. A minimum time threshold was used to remove respondents who rushed through responses. Duplicate respondents were also removed.

When designing our survey, we initially considered classifying exposure to flavor restrictions based on state of residence. However, this presented two issues: 1) enforcement issues meant that some people living in a state with a restriction may not have experienced a change in access to flavored e-cigarettes; 2) some people living in states without a restriction may have still been exposed to a restriction at the local level. Because reliance solely on state of residence leads to misclassification of exposure, we concluded that the most accurate way to determine whether someone experienced a loss of retailer access to flavored e-cigarettes would be to directly ask them in the survey. See survey questions below.

The sampling design was constructed to increase the likelihood that participants had experienced real-world restrictions on flavored e-cigarettes. E-cigarette flavor restrictions occurred in the context of state policies that raised the minimum age of tobacco purchase to 21, so all 50 states and Washington DC were categorized into one of three state groups with distinct tobacco control policy environments. Group 1 comprised of ‘early adopter’ states with e-cigarette flavor restrictions in place at the time of data collection in 2021. Group 2 comprised of states with a Tobacco-21 law prior to the federal Tobacco-21 law but no state-wide flavor restriction in effect. The remaining states in Group 3 did not have a state-enacted Tobacco-21 law in effect prior to the Federal law. (Table S1).

Survey quotas were designed so that individuals in Group 1 were oversampled, with quotas for a more representative sample of people who used e-cigarettes ages 18-32 according to gender (men, women), race (Black, non-Black), age (18-22, 23-27 and 28-32), and education (high school degree or less vs. some college or more). Subgroup quotas in each group were based on prevalence estimates in the Tobacco Use Supplement to the Current Population Survey (TUS-CPS) 2018-2019 data. To ensure a sufficient sample of participants who smoked cigarettes, the age limit was relaxed to include adults ages 33-41, although the present analysis is restricted to ages 18-34 to focus on young adults.

*Subgroup analyses.* For interested readers, subgroup estimates disaggregated by gender (women, men), age (18-34, 35-41), race/ethnicity (Black non-Hispanic, White non-Hispanic, Hispanic), and education (less than a college degree, college degree or more) are available as a supplemental Excel file.

The study was approved by the Yale Human Subjects Committee and University of Michigan Institutional Review Board Health Sciences and Behavioral Sciences (HUM #00190165) and exempt from ongoing oversight.

**Inclusion criteria**

*Sample.* Among the 1,961 adult respondents (ages 18-34), 1,025 used e-cigarettes exclusively and 936 dual used e-cigarettes and cigarettes. Of the 1,961 respondents, 827 of them were individuals who dual use cigarettes and e-cigarettes recruited through a similar smoking survey. These respondents were asked the same exact questions, but questions about cigarette smoking came first in the survey before questions about vaping. Because Tobacco 21 laws may have impacted some individuals’ ability to purchase their usual products, we asked respondents to select reasons why they were unable to purchase them. Those who indicated that “I was under age 21 at the time, too young to buy” were excluded from the analysis. The sample was finally restricted to individuals who reported residing in the same state as they did on Thanksgiving 2019, were unaffected by Tobacco 21 laws, and reported that they used flavored e-cigarettes. Respondents were asked “In the 30 days before Thanksgiving 2019, on how many days do you think you used e-cigarettes?” Individuals who reported using on ≥7 days were included in the study. After exclusions, the final sample size was 1,253 individuals.

*Allocation to real-world vs hypothetical flavor ban survey.* Participants were then given the following prompt: “In this survey, the term flavored e-cigarettes refers to all flavors except for tobacco. Examples of flavored e-cigarettes include those that are menthol/mint, fruit, dessert, candy or other sweets, or alcohol flavored. Do you ever remember a time when you first found that you could not buy flavored e-cigarettes (e.g., menthol/mint, fruit, candy, others) at a retail store or online as usual?” Those who responded with “Yes, I remember” were assigned the Real-World Flavored E-cigarette Ban questions, while those who responded “No, I do not remember” were assigned to the Hypothetical Flavored E-cigarette Ban. Individuals who stated that they do not use flavored e-cigarettes were excluded.

JUUL voluntarily removed specific flavors from the market, which we consider having the same effect on consumer purchasing as a ban. Therefore, those who responded by saying “Flavored e-cigarettes were not available at retail stores”, “JUUL was no longer selling its flavors in stores”, or “JUUL was no longer selling my preferred flavor online”, were included in the analysis and considered to have been affected by a flavor ban.

Respondents also had the option to write in responses. We reviewed and re-coded these accordingly: respondents who wrote in reasons such as “county ban”, “Governor enacted ban”, “Massachusetts banned flavors”, “temporary ban in my state”, “my state banned flavors” were re-coded and included in the group affected by flavor restrictions, while those who wrote in other responses like “out of stock” were excluded.

All analyses were conducted in R.

**Table S1. State groups by tobacco control policy environment**

| **GROUP 1**  **State-enacted permanent flavor restrictions on e-cigarettes as of March 2021** | **GROUP 2**  **State-enacted Tobacco-21 Law BEFORE the federal Tobacco-21 law and NO permanent flavor restrictions on e-cigarettes as of March 2021** | **GROUP 3**  **NO state-enacted Tobacco-21 Law BEFORE the federal Tobacco-21 law** |
| --- | --- | --- |
| 1. Massachusetts (flavor restriction effective 11/27/19 for e-cigarettes; 6/1/20 for all other products; T21 effective 12/31/18) 2. New Jersey (flavor restriction effective 4/20/20; T21 effective 11/1/17) 3. New York (flavor restriction effective 5/18/20; T21 effective 11/13/19) 4. Rhode Island (flavor restriction effective 3/26/20; T21 effective 7/1/21) | 1. Hawaii (effective 1/1/2016) 2. District of Columbia (effective 10/1/2018) 3. California (effective 6/9/2016) 4. Oregon (effective 1/1/2018) 5. Maine (effective 7/1/2018) 6. Illinois (effective 7/1/2019) 7. Virginia (effective 7/1/2019) 8. Delaware (effective 7/16/2019) 9. Arkansas (effective 9/1/2019) 10. Texas (effective 9/1/2019) 11. Vermont (effective 9/1/2019) 12. Connecticut (effective 10/1/2019) 13. Maryland (effective 10/1/2019) 14. Ohio (effective 10/17/2019) | 1. Alabama 2. Alaska 3. Arizona 4. Florida 5. Idaho 6. Kansas 7. Louisiana 8. Michigan 9. Missouri 10. Montana 11. Nevada 12. North Carolina 13. North Dakota 14. South Carolina 15. West Virginia 16. Wisconsin 17. Washington 18. Oklahoma 19. Iowa 20. Indiana 21. Pennsylvania 22. South Dakota 23. Utah 24. Wyoming 25. Mississippi 26. Colorado 27. New Hampshire 28. Georgia 29. Kentucky 30. Minnesota 31. Nebraska 32. New Mexico 33. Tennessee |

Notes: States were assigned to groups based on state-wide policy as of 2021 when the survey was disseminated. The Federal Tobacco-21 law went into effect on 12/20/2019, so states that implemented Tobacco-21 laws after this date were assigned to group 3. Montana and Washington both imposed emergency flavor restrictions on e-cigarettes that were temporary and expired in 2020 (Montana: 12/2019-4/2020; Washington: 10/2019-2/2020).

**Figure S1. E-cigarette flavor restrictions survey flowchart and sample inclusion criteria**

Do you ever remember a time when you first found that you could not buy flavored e-cigarettes (e.g. menthol/mint, fruit, candy, others) at a retail store or online as usual?

No, I do not use flavored
e-cigarettes
N = 241

Dual use on Thanksgiving 2019

N = 109 + 827 = 936

**Hypothetical Flavored E-cigarette Ban**

N = 503

**Real World Flavored
E-cigarette Restrictions**

N = 750

During the time when you were unable to purchase these products, why were you unable to purchase them?*

I was under age 21 at the time,

I don’t know,

Something else*

N = 338

**Exclusions**

Exclusive vaping on Thanksgiving 2019

N = 1,025

In the 30 days before Thanksgiving 2019, on how many days do you think you used e-cigarettes? (0-30)

Have you smoked at least 100 cigarettes in your entire life?

Which state do you currently live in now?

Did you live in this same state on Thanksgiving 2019?

I do not currently reside in the U.S.

No

No,

Don’t know

<7 days

<7 days

N= 129

**Vaping Survey**

N = 1,134

**Smoking Survey**

N = 827

In the 30 days before Thanksgiving 2019, on how many days do you think you smoked cigarettes? (0-30)

On how many of the past 30 days have you used e-cigarettes?

*Respondents were prompted to select all that apply. Those who selected “Something else (please specify)” and wrote responses that referred to flavor restrictions were included. Any responses that included “I was under 21 at the time” were excluded.

**Table S2. Real-world e-cigarette flavor restrictions survey, sample characteristics**

| **Real-world e-cigarette flavor restrictions survey** | **Exclusive e-cigarette use on Thanksgiving 2019** | | **Dual use on Thanksgiving 2019** | |
| --- | --- | --- | --- | --- |
|  | **N= 281** | **weighted %** | **N = 469** | **weighted %** |
|  |  |  |  |  |
| **Age** |  |  |  |  |
| 18-21 | 46 | 13.1% | 34 | 8.3% |
| 22-24 | 86 | 29.1% | 102 | 24.2% |
| 25-29 | 79 | 32.3% | 183 | 37.6% |
| 30-34 | 70 | 25.5% | 150 | 29.9% |
| **Gender** |  |  |  |  |
| Man | 129 | 30.9% | 253 | 46.2% |
| Woman | 141 | 65.0% | 197 | 48.2% |
| Non-binary / Prefer not to say | 11 | 4.1% | 19 | 5.6% |
| **Race/Hispanic origin** |  |  |  |  |
| Hispanic | 35 | 11.7% | 85 | 18.3% |
| Black, non-Hispanic | 13 | 10.0% | 24 | 13.2% |
| White, non-Hispanic | 209 | 70.6% | 335 | 63.1% |
| Other, non-Hispanic | 24 | 7.7% | 25 | 5.4% |
| **Education** |  |  |  |  |
| High school degree or Less | 82 | 24.0% | 159 | 26.1% |
| Some college | 87 | 30.7% | 124 | 34.7% |
| At least college degree | 112 | 45.2% | 186 | 39.2% |
| **Census region** |  |  |  |  |
| Northeast | 153 | 57.5% | 319 | 70.3% |
| Midwest | 26 | 8.1% | 33 | 5.9% |
| South | 39 | 12.6% | 34 | 6.6% |
| West | 63 | 21.8% | 83 | 17.3% |
| **State group** |  |  |  |  |
| Group 1 | 139 | 53.9% | 307 | 68.3% |
| Group 2 | 57 | 21.0% | 72 | 14.6% |
| Group 3 | 85 | 25.1% | 90 | 17.1% |
| **E-cigarette use in 30 days pre-Thanksgiving 2019** |  |  |  |  |
| 0 days | 0 | 0% | 0 | 0% |
| 1-6 days | 0 | 0% | 0 | 0% |
| 7-10 days | 17 | 5.8% | 83 | 21.2% |
| 11-20 days | 66 | 25% | 139 | 30.8% |
| 21-30 days | 198 | 69.2% | 247 | 48.0% |
| **Cigarette smoking in 30 days pre-Thanksgiving 2019** |  |  |  |  |
| 0 days | 281 | 100% | 0 | 0% |
| 1-6 days | 0 | 0% | 145 | 42.5% |
| 7-10 days | 0 | 0% | 53 | 11.6% |
| 11-20 days | 0 | 0% | 86 | 15.6% |
| 21-30 days | 0 | 0% | 185 | 30.3% |

**Table S3. Hypothetical federal e-cigarette flavor ban survey, sample characteristics**

| **Hypothetical e-cigarette flavor restrictions survey** | **Exclusive e-cigarette use*** | | **Dual use*** | |
| --- | --- | --- | --- | --- |
|  | **N= 158** | **weighted %** | **N= 345** | **weighted %** |
|  |  |  |  |  |
| **Age** |  |  |  |  |
| 18-21 | 39 | 20.0% | 40 | 15.3% |
| 22-24 | 38 | 22.3% | 49 | 13.2% |
| 25-29 | 45 | 32.4% | 121 | 30.7% |
| 30-34 | 36 | 25.3% | 135 | 40.8% |
| **Gender** |  |  |  |  |
| Man | 75 | 33.5% | 174 | 41.3% |
| Woman | 77 | 62.2% | 156 | 54.1% |
| Non-binary / Prefer not to say | 6 | 4.2% | 15 | 4.6% |
| **Race/Hispanic origin** |  |  |  |  |
| Hispanic | 21 | 11.6% | 53 | 16.9% |
| Black, non-Hispanic | 7 | 9.6% | 24 | 8.0% |
| White, non-Hispanic | 117 | 70.1% | 244 | 68.1% |
| Other, non-Hispanic | 13 | 8.8% | 24 | 6.9% |
| **Education** |  |  |  |  |
| High school degree or Less | 54 | 24.2% | 129 | 25.0% |
| Some college | 59 | 43.4% | 89 | 32.0% |
| At least college degree | 45 | 32.3% | 127 | 43.0% |
| **Census region** |  |  |  |  |
| Northeast | 55 | 36.3% | 197 | 62.6% |
| Midwest | 20 | 8.5% | 44 | 9.6% |
| South | 31 | 22.8% | 32 | 8.1% |
| West | 52 | 32.4% | 72 | 19.8% |
| **State group** |  |  |  |  |
| Group 1 | 50 | 32.9% | 187 | 59.9% |
| Group 2 | 53 | 37.4% | 58 | 17.4% |
| Group 3 | 55 | 29.8% | 100 | 22.7% |
| **E-cigarette use in past 30 days*** |  |  |  |  |
| 0 days | 0 | 0% | 0 | 0% |
| 1-6 days | 0 | 0% | 0 | 0% |
| 7-15 days | 35 | 20.8% | 132 | 35.8% |
| 16-30 days | 123 | 79.2% | 213 | 64.2% |
| **Cigarette smoking in past 30 days*** |  |  |  |  |
| 0 days | 158 | 100% | 8 | 2.0% |
| 1-6 days | 0 | 0% | 6 | 4.5% |
| 7-15 days | 0 | 0% | 56 | 21.8% |
| 16-30 days | 0 | 0% | 203 | 71.7% |
| **Nicotine dependence*  (Time to first tobacco use after waking up)** |  |  |  |  |
| Within 5 minutes | 36 | 23.8% | 86 | 21.2% |
| 6 to 30 minutes | 39 | 24.3% | 112 | 30.5% |
| 31 to 60 minutes | 37 | 21.2% | 81 | 23.2% |
| More than 1 hour | 46 | 30.7% | 66 | 25.1% |

*Note: These survey questions reflect tobacco use at time of survey, and not on Thanksgiving 2019: “On how many of the past 30 days have you used e-cigarettes?”, “On how many of the past 30 days have you smoked cigarettes?”, “On the days that you *smoke now*, how soon after you wake up do you typically smoke *your first cigarette of the day*?” and “On the days that you *smoke now*, how soon after you wake up do you typically smoke *your first cigarette of the day*?”

**Real-world Flavor Ban Responses**

Q38 In this survey, the term flavored e-cigarettes refers to all flavors except for tobacco. Examples of flavored e-cigarettes include those that are menthol/mint, fruit, dessert, candy or other sweets, or alcohol flavored.

Do you ever remember a time when you first found that you could not buy flavored e-cigarettes (e.g. menthol/mint, fruit, candy, others) at a retail store or online as usual?

- Yes, I remember
- No, I do not remember
- No, I do not use flavored e-cigarettes

Q42 During the time when you were not able to purchase these products, why were you unable to purchase them? Select all that apply.

- Flavored e-cigarettes were not available at retail stores
- I was under age 21 at the time, too young to buy.
- JUUL was no longer selling its flavors in stores.
- JUUL was no longer selling my preferred flavor online.
- Something else (please specify) ________________________________________________
- I don’t know

Q43 After this change when you could not purchase *flavored e-cigarettes* (e.g. menthol/mint, fruit, candy, others), what did you do? Select all that apply.

- Switch to using tobacco flavored e-cigarettes
- Switch to smoking cigarettes or other combustible tobacco (e.g. cigars, hookah, pipe tobacco, bidis)
- Switch from JUUL to other flavored e-cigarettes.
- Switch to using smokeless tobacco (e.g. chewing tobacco, snus, snuff, dip, dissolvables)
- Switch to using heated tobacco (e.g. IQOS, Eclipse)
- Quit all vaping and tobacco use
- Continued vaping flavored e-cigarettes by getting them from a different source
- Continued vaping by buying with a fake ID

[If “Continue vaping flavored e-cigarettes by getting them from a different source”]

Q44 How were you able to continue obtaining *flavored e-cigarettes* (e.g. menthol/mint, fruit, candy, others)? Select all that apply.

- Purchased from another state, locality, or country
- Purchased online
- Obtained through family and friends
- Modified e-cigarettes by adding my own flavors
- Purchased from the black market
- Other

Q49 After this change, did your perceptions of the health risks of vaping e-cigarettes change?

- Yes, it seemed riskier to vape
- Yes, it seemed less risky
- No

Q50 After this change, did your perceptions of the health risks of vaping *flavored e-cigarettes* in particular change?

- Yes, it seemed riskier to vape flavored e-cigarettes
- Yes, it seemed less risky
- No

**Hypothetical Flavor Ban Scenario**

Q51 **Reminder:** In this survey, the term ***flavored e-cigarettes*** refers to all flavors except for tobacco. Examples of flavored e-cigarettes include those that are menthol/mint, fruit, dessert, candy or other sweets, or alcohol flavored.
 
Suppose the federal government bans sales of all ***flavored e-cigarettes*** in the U.S. and you are no longer able to purchase them through your usual source. What do you think you would do? Select all that apply.

- Switch to vaping tobacco-flavored e-cigarettes
- Switch to smoking menthol cigarettes
- Switch to smoking non-menthol cigarettes or other combustible tobacco (e.g. cigars, hookah, pipe tobacco, bidis)
- Switch to using smokeless tobacco (e.g. chewing tobacco, snus, snuff, dip, dissolvables)
- Switch to using heated tobacco (e.g. IQOS, Eclipse)
- Quit all vaping and tobacco use
- Continue vaping flavored e-cigarettes by getting them from a different source
- Nothing -- I do not vape flavored e-cigarettes

[If “Continue vaping flavored e-cigarettes by getting them from a different source”]

Q52 How would you try to continue obtaining flavored e-cigarettes? Select all that apply.

- Purchase from another country
- Purchase online
- Get through family and friends
- Modify e-cigarettes on my own (adding own flavors)
- Purchase from the black market
- Other

**Table S4. Continued vaping of flavored e-cigarettes following a ban among US young adults who use flavored e-cigarettes.**

| Real-world flavored e-cigarette ban | | | Hypothetical federal flavored e-cigarette ban | | |
| --- | --- | --- | --- | --- | --- |
| How were you able to continue obtaining flavored e-cigarettes? Select all that apply. | n | % (95% CI) | How would you try to continue obtaining flavored e-cigarettes? Select all that apply. | n | %  (95% CI) |
| Purchased from the black market | 31 | 8.2 (4.7, 11.7) | Purchase from the black market | 33 | 16.6 (10.3, 22.9) |
| Purchased from another state, locality, or country | 130 | 44.6 (37.7, 51.5) | Purchase from another country | 57 | 32.1 (23.5, 40.7) |
| Purchased online | 145 | 50.3 (43.4,57.2) | Purchase online | 160 | 77.1 (69.2, 85.1) |
| Obtained through family and friends | 96 | 30.1 (24, 36.2) | Get through family and friends | 82 | 45.5 36.8, 54.2) |
| Modified e-cigarettes by adding my own flavors | 41 | 11.7 (7.4, 16.0) | Modify e-cigarettes on my own (adding own flavors) | 39 | 22.3 (14.3, 30.2) |
| Other | 36 | 11.6 (7.3, 15.9) | Other | 8 | 2.8 (0.4, 5.2) |

Notes: People who use flavored e-cigarettes and reported that they would ‘continue vaping flavored e-cigarettes by getting them from a different source’, following a hypothetical federal e-cigarette flavor cigarette ban (n=199) or who reported that they ‘continued vaping flavored e-cigarettes by getting them from a different source’, following real-world flavored e-cigarette bans (n=298). Numbers do not sum to 100% because respondents were allowed to select more than one option.

**Table S5. Responses to hypothetical federal flavored e-cigarette ban among US young adults who vape flavored e-cigarettes: Menthol vs. Non-menthol cigarette use.**

| Young adults who use flavored e-cigarettes  n=431 | Young adults who exclusively use e-cigarettes, n= 158 | | Young adults who dual use, n=273 | |
| --- | --- | --- | --- | --- |
| Response options | n | %  (95% CI) | n | %  (95% CI) |
| Switch to vaping tobacco-flavored e-cigarettes | 35 | 22.1 (14.1, 30.1) | 116 | 34.6 (28.2, 41.0) |
| Switch to smoking menthol cigarettes | 18 | 11.2 (5.7, 16.7) | 106 | 32.4 (26.2, 38.6) |
| Switch to smoking non-menthol cigarettes or other combustible tobacco (e.g. cigars, hookah, pipe tobacco, bidis) | 17 | 12.5 (5, 20) | 56 | 17.4 (12.2, 22.6) |
| Switch to using smokeless tobacco (e.g. chewing tobacco, snus, snuff, dip, dissolvables) | 7 | 5.3 (-0.2, 10.7) | 34 | 9.5 (5.9, 13.1) |
| Switch to using heated tobacco (e.g. IQOS, Eclipse) | 2 | 1.8 (-0.8, 4.4) | 30 | 8.7 (5.0, 12.4) |
| Quit all vaping and tobacco use | 53 | 34.5 (26, 43) | 63 | 17.2 (12.4, 22.0) |
| Continue vaping flavored e-cigarettes by getting them from a different source | 76 | 49.3 (40.2, 58.4) | 123 | 38.0 (31.4, 44.6) |

Notes: Numbers do not sum to 100% because respondents were allowed to select more than one option.
